# The GH-IGF-1 Axis in Circadian Rhythm

**DOI:** 10.3389/fnmol.2021.742294

**Published:** 2021-09-09

**Authors:** Weihao Wang, Xiaoye Duan, Zhengxiang Huang, Qi Pan, Chen Chen, Lixin Guo

**Affiliations:** ^1^Department of Endocrinology, Beijing Hospital, National Center of Gerontology, Institute of Geriatric Medicine, Chinese Academy of Medical Sciences, Beijing, China; ^2^School of Biomedical Sciences, University of Queensland, Brisbane, QLD, Australia

**Keywords:** GH, IGF-1, metabolism, clock, circadian rhythm

## Abstract

Organisms have developed common behavioral and physiological adaptations to the influence of the day/night cycle. The CLOCK system forms an internal circadian rhythm in the suprachiasmatic nucleus (SCN) during light/dark input. The SCN may synchronize the growth hormone (GH) secretion rhythm with the dimming cycle through somatostatin neurons, and the change of the clock system may be related to the pulsatile release of GH. The GH—insulin-like growth factor 1 (IGF-1) axis and clock system may interact further on the metabolism through regulatory pathways in peripheral organs. We have summarized the current clinical and animal evidence on the interaction of clock systems with the GH—IGF-1 axis and discussed their effects on metabolism.

## Introduction

All animals live under the influence of the 24-h cycle of the earth’s rotation. Organisms sense these regular external changes and synchronize their physical activities, such as behavior, food intake, energy metabolism, sleep, reproductive activity, and immune function, to increase their survival abilities (Takahashi et al., 2008). Organisms have developed a highly conservative and complex molecular clock system, which creates an internal circadian rhythm during light/dark input (Buhr and Takahashi, 2013). The output of this regulatory system is linked to numerous organs and tissues, relaying different signals released by the central circadian system (Hastings et al., 2007). The mammalian brain’s central clock system consists of pairs of SCN which locate at the base of the hypothalamus. These clusters of about 10,000 GABA-enabled neurons including ventricular cores that receive direct neural control from the retina and brainstem region (Reppert and Weaver, 2002). SCN controls the endocrine cycle and metabolic rhythm in two ways. First, SCN determines the timing of sleep-dependent events, such as nocturnal secretions of prolactin and growth hormone, by dissecting the centers that control sleep and wakefulness. In addition, the central clock system can regulate the rhythmic release of hormones such as melatonin and cortisol by linking to the neuroendocrine and autonomic nervous systems independently of sleep-driving hormones and other rhythms (Hastings et al., 2007). The peripheral clock system plays a role in almost all organs and tissues. The activity of the peripheral clock system is synchronized with the central master clock system through body fluids and neural connections. The central and peripheral clocks use the same set of transcription factors, including CLOCK and BMAL1, to generate circadian pattern of gene expression (Kalsbeek et al., 2006; Nicolaides et al., 2014).

SCN may act on somatostatinergic neurons and GH-releasing hormones (GHRH) to synchronize the GH rhythm with the light-dark cycle (Willoughby and Martin, 1978; Vaccarino et al., 1995; Davies et al., 2004). At the same time, as the aging process progresses, the decrease in the clock rhythm (Hastings et al., 2003) is also consistent with the decrease in the pulsatile release of GH (Kuwahara et al., 2004). Therefore, there may be a connection between the clock system and the rhythmic release of hormones. A large number of experiments and reviews have confirmed the interaction between circadian rhythm regulation and the hypothalamus-pituitary-adrenal cortisol (HPA) axis (Nader et al., 2010; Nicolaides et al., 2014). There is currently a lack of relevant review to summarize the interaction between circadian rhythm regulation and the GH/IGF-1 axis. Our previous experiments have confirmed that the circadian rhythm disorder caused by changes in lighting interferes with the pulsatile release pattern of growth hormone in male mice and is accompanied by changes in the expression of peripheral clock genes in the liver (Wang et al., 2021). Therefore, this review will focus on the interaction between the circadian rhythm and the GH-IGF-1 axis and its effect on metabolism.

## Circadian Clock System

**FIGURE 1:**
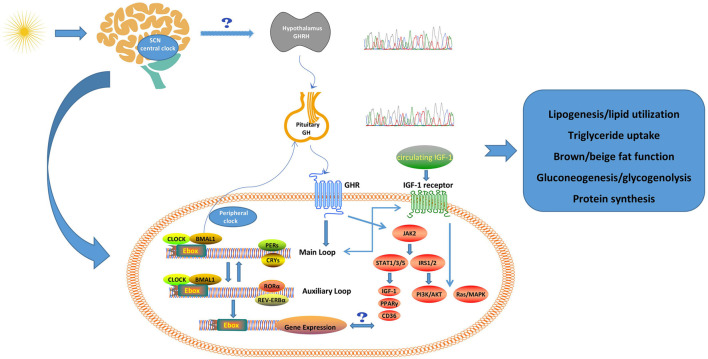
Possible crosstalk between clock system and GH/IGF-1 axis. The CLOCK system forms an internal circadian rhythm in the SCN during light/dark cycles. The SCN may synchronize the GH secretion rhythm with the dimming cycle through somatostatin neurons, and the change of the clock system may be related to the pulsatile release of GH. The GH/IGF-1 axis and clock system may interact further on the metabolism through several pathways in peripheral tissues (e.g., liver, fat). The physiological roles of GH and IGF-1 are also summarized in this figure (Huang et al., 2020b). SCN, suprachiasmatic nucleus; GHRH, Growth hormone-releasing hormone; GH, growth hormone; GHR, growth hormone receptor; IGF-1, insulin-like growth factor 1; BMAL1, brain-muscle-arnt-like protein 1; PERs, periods; CRYS, Cryptochromes; RORα, retinoic acid-related orphan receptor α; Ebox, enhancer motif; JAK2, Janus kinase 2; STAT, signal transducer and activator of transcription; IRS, insulin receptor substrate; PPARγ, peroxisome proliferator-activated receptor γ; PI3K/Akt, phosphatidylinositol 3-kinase/protein kinase B.

The CLOCK transcript forms a heterodimer with brain and muscle arnt-like protein 1 (BMAL1). Under the control of the biological clock system, the heterodimer CLOCK/BMAL1 and a series of other transcription factors are responsible for the circadian oscillation of gene expression (Hastings et al., 2007; Takahashi et al., 2008). The molecular mechanism of the circadian oscillation of gene expression is mediated by the transcription/translation feedback loop (Nader et al., 2010). The CLOCK/BMAL1 heterodimer combines with the E-box response element which located in the promoter region to stimulate the expression of other target genes, the core of which is the transcriptional expression of other clock genes, such as Periods (*PER1*, *PER2*, and *PER3*), and Cryptochromes (*CRY1* and *CRY2*). The activated *Pers* and *Crys* stimulate the activities of casein kinases 1ε/δ and inhibit the transcriptional activity of the CLOCK/BMAL1 by inhibiting the binding to the E-box response element. A negative feedback transcription cycle is eventually formed to maintain the oscillation of the gene expression (Kiyohara et al., 2006; Kondratov et al., 2006b). In addition to the regulation of this main transcription loop, CLOCK/BMAL1 stimulates the expression of other clock-related transcriptions, such as *REV-ERB*α, retinoic acid-borne orphaned binders a (*ROR*α), *DEC1*, *DEC2*, and albumin D-binding (*DBP*), which form an auxiliary loop that stabilizes the main regulatory loop (Ripperger and Schibler, 2006; Padmanabhan et al., 2012). Importantly, transcription factors in the main regulatory and auxiliary loops control many downstream circadian clock-related genes and affect a variety of biological activities such as sleep/wake cycle, eating pattern, energy consumption, and glucose metabolism (Takahashi et al., 2008; Figure 1). In addition to neural connections, the central clock system also synchronizes the circadian rhythms of the peripheral clock system through hormones or factors, such as arginine vasopressin (AVP) and tumor necrosis factor (TNF)α (Kraves and Weitz, 2006; Hastings et al., 2007).

The clock system mainly regulates metabolism in the following three ways. The first one is to control nuclear receptors. The turnover of carbohydrates, proteins, and lipids, and the production/storage of energy are necessary for survival. Approximately 10% of energy-controlling gene expression, including those encoding nuclear hormone receptors and glucose and lipid metabolism enzymes, are regulated by circadian rhythm in a tissue-specific manner (Panda et al., 2002; Storch et al., 2002; Yang et al., 2006). Nuclear receptors constitute a superfamily of ligand-activated transcription factors, which regulate critical physiological processes including growth, development, hormonal signals, reproduction, and energy metabolism (Sonoda et al., 2008). Special nuclear receptors are used as sensors for metabolites such as hormones, vitamins, and lipids. The expression of some nuclear receptors is regulated by CLOCK and BMAL1. These receptors include retinoic acid-related orphan receptor α (RORα), REV-ERBα, and peroxisome proliferator-activated receptor (PPAR)α (Oishi et al., 2005). One of the nuclear receptors, REV-ERBα, is also a negative regulator of the rhythmic CLOCK transcription circuit. It may inhibit glucogenesis, lipid metabolism, adipocyte differentiation, and the transcriptional activities of several other nuclear receptors, including PPARγ and RORα (Yin et al., 2007; Duez and Staels, 2008; Figure 1). CLOCK−/− and BMAL1−/− mice exhibit disorders of glucose metabolism and circadian changes in circulating glucose and triglycerides, which lead to obesity, hyperlipidemia, and diabetes (Rudic et al., 2004; Turek et al., 2005; Sahar and Sassone-Corsi, 2012; Albrecht, 2017). The mice have increased expression of plasminogen activator inhibitor-1 (PAI-1), which is a known risk factor for obesity, diabetes, and cardiovascular disease (Oishi et al., 2006; Oishi, 2009). Another circadian clock protein PER2 inhibits the expression of PAI-1 in a CLOCK/BMAL1-dependent manner as an important factor in the development of these metabolic diseases after the circadian clock system is dysregulated (Oishi et al., 2009). In addition, the mRNA expression of *BMAL1*, *PER2*, and *CRY1* in visceral fat is closely related to the increase in waist circumference which is an indicator of metabolic syndrome (Gómez-Abellán et al., 2008).

The second is that the circadian clock system may control the rate-limiting steps of the metabolic process (Panda et al., 2002). For example, the activation of the rate-limiting enzyme HMG-CoA reductase (HMGCR) in cholesterol biosynthesis shows circadian rhythm (Demierre et al., 2005), and the activity is the highest during the night. In addition, the circadian clock system may control the expression of nicotinamide phosphosarcosyltransferase (NAMPT), which is a key rate-limiting enzyme in the salvage pathway of NAD^+^ biosynthesis (Ramsey et al., 2009). The rhythm of the enzyme expression drives the oscillation of NAD + levels, and the synthesis of NAD^+^ is involved in the process of aging and lipid metabolism (Belenky et al., 2007). NAD^+^ regulates the circadian clock system through SIRT1. SIRT1 is a histone deacetylase, which may regulate the transcriptional activity of BMAL1/CLOCK, forming a metabolic feedback loop again between the circadian clock system and metabolism (Nakahata et al., 2009; Ramsey et al., 2009).

The last pathway is that the circadian clock system controls cell metabolism by regulating the nutrient sensors Sirt1 and AMP-activated protease (AMPK) (Sahar and Sassone-Corsi, 2012). Sirt1 regulates gene expression through histone deacetylation. Circadian gene expression and BMAL1 acetylation are disturbed in liver-specific SIRT1 mutant mice (Nakahata et al., 2008). AMPK is a key factor in energy regulation. The activity of AMPK is found to be rhythmic in mouse liver, hypothalamus, and fibroblasts (Lamia et al., 2009). AMPK may regulate the circadian rhythm by phosphorylating CRY1 (Lamia et al., 2009) and casein kinase 1 (CK1)ε (Yang et al., 2017). CK1ε plays an important role in regulating the circadian rhythm by phosphorylating PER protein and controlling its degradation (Shirogane et al., 2005). Interestingly, the activation of AMPK also leads to an increase in NAD^+^ levels (Cantó et al., 2009). Therefore, AMPK may indirectly regulate the expression of circadian genes through the activation of SIRT1.

The circadian rhythm system, *CLOCK* gene mechanism and metabolic pathways are intertwined by neural circuits which transmit the environmental signals to peripheral organs through hormones, chemokines and neuropeptides (Mazzoccoli et al., 2012). The desynchronization among central and peripheral clock system, metabolic pathways and regulators may impair the metabolic homeostasis which could contribute to the progress of obesity, metabolic syndrome, and diabetes.

## GH-IGF-1 Axis

One major function of GH is to stimulate tissue growth. Lack of GH may lead to dwarfism, while excessive GH may lead to giantism. A variety of neurotransmitter pathways, as well as various peripheral feedback signals, regulate the secretion of GH by acting directly on the anterior pituitary gland and/or by regulating the release of GHRH or somatostatin in the hypothalamus (Giustina and Veldhuis, 1998). GH secreted from the pituitary gland acts on the peripheral organs and stimulates the production of IGF-1 (Cuttler, 1996). Growth hormone and IGF-1 play a variety of regulatory roles in the body. One of the major functions of GH and IGF-1 is to promote linear growth. However, GH and IGF-1 have different effects on glucose and lipid metabolism. GH may antagonize some actions of insulin, to promote lipolysis and hinder adipogenesis, while IGF-1 has the opposite effect (Moøller and Joørgensen, 2009). In the feeding state, GH secretion decreases while insulin secretion increases, leading to increased glucose uptake by skeletal muscle and fat accumulation. In the fasting state, GH increases lipolysis and hepatic glucose output while insulin concentration decreases (Rabinowitz et al., 1965). However, a meta-analysis revealed that fasting and energy restricting diets did not generate a significant effect on circulating IGF-1 (Rahmani et al., 2019). A concept of insulin-growth hormone balance has been proposed that the ratio of two hormones is closely related to the glucose and lipid metabolism and energy metabolism of obese patients (Huang et al., 2020b). After adulthood, the secretion of GH and IGF-1 continues to decrease, and the secretion of elderly people over 60 years old is significantly diminished (Zadik et al., 1985). Studies have revealed that the GH/IGF-1 axis plays a key role in the aging process of humans and animals (Junnila et al., 2013). At the same time, the GH/IGF-1 axis is also involved in the pathogenesis of obesity (Berryman et al., 2013), cardiovascular disease (Colao, 2008), and tumor (Chhabra et al., 2011).

GH receptor is a member of the class I cytokine receptor family and exists in almost all cell types in the human body (Waters et al., 1999). GH activates Janus kinase 2 (JAK2)/STAT and Src/MAPK pathways after binding to GH receptor (Brooks and Waters, 2010). The former mainly regulates metabolism, while the latter regulates mitotic function. JAK2 controls the metabolic effects by activating STAT1, 3, and 5, of which STAT5 is the most prominent one. It may also promote the production of IGF-1 to accelerate linear growth (Woelfle et al., 2003). Studies have shown that JAK2 may also phosphorylate insulin receptor substrate 1/2 (IRS1/2) and activate the phosphatidylinositol 3-kinase/protein kinase B (PI3K/AKT) pathway (Ridderstrale et al., 1995; Argetsinger et al., 1996; Yamauchi et al., 1998; Figure 1). However, these studies either used super-physiological GH doses (Ridderstrale et al., 1995; Yamauchi et al., 1998) or did not evaluate the physiological effects of GH administration (Argetsinger et al., 1996), and the conclusions may not apply to physiological situations. Another study has shown that GH increases lipolysis by activating MEK/ERK and inhibiting PPARγ and fat-specific protein 27 (FSP27) (Sharma et al., 2019). GH also stimulates muscle lipid uptake by increasing muscle lipoprotein lipase (LPL) activity (LeRoith and Yakar, 2007). The free fatty acids released from white adipose tissue (WAT) are absorbed and oxidized in other tissues, so the net effect of elevated GH promotes the reduction of body fat accumulation. Recent studies have also found that the GHR-JAK2-STAT5 signal inhibits lipid uptake and neo-adipogenesis in the liver, partly by inhibiting PPARγ and downstream CD36 (Sos et al., 2011; Liu et al., 2016; Chhabra et al., 2019). This shows that GH has a direct effect on lipid metabolism in the liver and may reduce the occurrence of non-alcoholic fatty liver.

Previous studies have revealed that GH controls the generation of IGF-1 by targeting the gene transcription of *IGF-1* via STAT5 in diverse physiological situations (Chia et al., 2010; Rotwein, 2012). The liver is the major organ of the synthesis of endocrine factors including IGF-1 and IGF-2, as well as the binding proteins (IGFBPs) (Adamek and Kasprzak, 2018). The secretion of IGF-1 and IGFBPs is not only under the control of endocrine and nutritional factors, but also autocrine and paracrine factors (Voci et al., 1999). The main signaling pathways downstream of the IGF-1 receptor are Ras/MAPK and PI3K/Akt, while these two pathways are responsible for the glucose and lipid metabolism in the liver (Huang et al., 2020b).

## Crosstalk Between Circadian Clock System and GH/IGF-1 Axis

### Regulatory Effect of the GH/IGF-1 Axis on the Circadian Clock System

There are very few reports to identify the regulatory effect of the GH/IGF-1 axis on the circadian clock system. One study raised an interesting point: circadian clock-related gene expression may exist in the pituitary, and this expression is related to the expression of GH gene (Kim et al., 2015). Fast-growing transgenic coho salmon including the OnMTGH1 gene construct was used as the target model (Kim et al., 2015). Vital clock genes in this research revealed various responses to the overexpression of GH. In this study, the correlation between the *CLOCK* gene and the *BMAL1* gene was not high, which was consistent with the view that different parts of the circadian clock system linked to different functions as circadian oscillators (Guillaumond et al., 2005). Most of the core clock genes (*CLOCK, PER1, PER2, CRY3, NRLD2*) showed the difference of expression oscillation in the pituitary of GH transgenic and wild type coho salmon, which indicated that the GH gene might have a regulatory effect on the expression of pituitary clock genes. There are several possible explanations for the different expression patterns (amplitude and phase) of core clock genes between transgenic and wild type coho salmon. GH regulates its own production in the pituitary through negative feedback control. This interference process may integrate metabolic processes in other parts of the body, such as the production of IGF-1 in the liver, to respond to changes in seasonal and nutritional challenges by affecting normal regulatory processes (Beckman, 2011). In addition, GH transgenic Coho salmon exhibited pathophysiological effects of increased GH expression, such as changes in reproduction, metabolism, stress, and disease resistance (Pitkänen et al., 1999; Bessey et al., 2004; Kim et al., 2013), which were all affected by the action of pituitary hormones, and might even be affected by changes in pituitary structure. It is known that overexpression of GH may affect the molting of coho salmon, which is a complex physiological process, and this process may affect the expression level, pattern, and/or effect of clock gene (Devlin et al., 2000). Although most studies have been carried out in vertebrates to determine the interaction between metabolism and clock genes (Delezie and Challet, 2011), transgenic GH over-expressing Coho salmon may be a useful model to improve the understanding of the interaction between circadian molecular clocks and nutritional status.

There are a few related human studies. Six patients with hypopituitarism were studied to determine the relationship between peripheral clock gene expression and GH (Sjögren et al., 2007). After 2 weeks of GH treatment (0.5 mg/day), systemic metabolic testing and skeletal muscle biopsy were performed in these patients. It was found that the plasma IGF-1 levels increased after GH treatment, accompanied by an increased expression of the *CLOCK* gene and decreased expression of the *PER1* gene in muscle tissues. This study suggested for the first time that GH might regulate the peripheral clock system by affecting the expression of *CLOCK* and *PERIOD* genes in humans. The most interesting observation was that there was an opposite effect on expression between the *CLOCK* gene and the *PER1* gene by GH. GH may be a potential medium for SCN to regulate the peripheral clock system. The rhythmic release of GH secreted by the pituitary gland is controlled by growth hormone-releasing hormone and somatostatin in the hypothalamus (Anderson et al., 2004). It may act as an endocrine factor to transmit signals from SCN to peripheral tissues.

### Regulation of Circadian Clock System on the GH/IGF-1 Axis

Human GH (hGH) transgenic mice were used to express the hGH gene in mouse pituitary somatotrophs (Vakili et al., 2016). This change of cell/tissue-specific expression was a result of a transgene containing the whole *hGH* gene and locus control region (LCR) in a fragment of human chromosome (Jin et al., 2009; Vakili et al., 2011, 2016). LCR was used as tissue or cell-specific enhancer and provided a suitable site for gene integration and independent expression. All mice presented normal growth patterns and specifically express (but not overexpress) of *hGH* gene in somatotrophs of the pituitary gland. Sequences of the *hGH* gene promoter revealed that the enhancer motif (Ebox) element could bind the circadian transcriptional regulators (CLOCK and BMAL1). In addition, CLOCK/BMAL1 was responsible for the transactivation of the hGH gene promotor. The article proves that the synthesis of hGH, especially the expression of the hGH gene, is under the control of circadian rhythm, and it is also the target of CLOCK/BMAL1 signals.

Previous studies reported that mice with defective clock genes [BMAL1−/− and CRY−/− (Cry1−/− and Cry2−/−)] had significantly slower growth and more weight loss (Masuki et al., 2005; Kondratov et al., 2006a; Sun et al., 2006; Bur et al., 2009). This growth defect became obvious 2–3 weeks after birth, which coincided with the maturation time of the somatotroph axis (Clark et al., 1985). It is well known that pulsatile release of GH may increase the synthesis of major urine proteins (MUP) in the liver of male mice and subsequent accumulation in the urine (Norstedt and Palmiter, 1984; Low et al., 2001). The study found that the main urine protein in the urine of CRY−/− male mice was reduced compared with that of wild-type mice, which was related to the downregulation of *MUP1* gene expression in the liver. This phenomenon was not observed in female CRY−/− mice, and the deletion of the *CRY* gene did not change the distribution of GH values in female mice. On the contrary, the random GH level of *CRY* gene-deficient male mice was significantly increased, and only 20% of the random GH values were lower than 1 ng/ml, indicating that the duration of the GH trough was shorter than that in the control group. These data revealed that the GH secretion profile of CRY−/− male mice were changed with reduced GH secretion. It was suggested that the expression of clock genes (such as *CRY*) was related to the pulsatile release of GH in male mice. Such change in male mice may contribute to metabolic sex dimorphism (Bur et al., 2009). In addition, the total content of GH in the pituitary of wild-type male mice is scattered in a wide range of values, which may indicate the ever-changing process of GH peaks and troughs. The secretion pattern with a low-amplitude irregular pulsatile profile in CRY−/− male mice was similar to that of female mice. This study also found that the *MUP1* mRNA levels were restored in CRY−/− male mice with the injection of bovine GH and octreotide (to inhibit endogenous GH secretion). The injection also reversed the feminization expression pattern of *CYP2B9, CYP2D9, CYP4A12, CYP7B1, ELOVL3* (dominant expression genes in female mice) in the liver to that of wild-type male mice. The results further confirmed the role of the GH axis in the sex dimorphism of CRY−/− male mice (Bur et al., 2009). A similar conclusion was reached in another study using genetically modified male rats (expressing human GH) as an experimental model (Hirao et al., 2010). Another hypothesis is that the circadian rhythm in the pituitary may synchronize the unitary ultradian activities of GH-secreting cells through a long-distance homotypic cell network (Bonnefont et al., 2000; Bonnefont and Mollard, 2003), although this possibility remains to be studied.

In addition, studies have confirmed that the disruption of the circadian clock system affects the release of GH and GH-mediated signal pathways. The BMAL1 knockout mouse (BMAL1−/−) model was used to explore the effects of circadian clock dysfunction on the GH circulating levels and downstream pathways (Lyu et al., 2020). In BMAL1−/− mice, the GH receptor (GHR) signaling was decreased, including reduced phosphorylation of GHR, JAK2, and STAT1/3/5. Such reduction might be due to the increased expression of the negative regulators, such as SOCS. Interestingly, the study tested the 24-h serum GH concentration of male and female mice (blood collection every 1 h) and found that there was no significant difference in serum GH concentration between BMAL1−/− mice and control mice. The level of IGF-1 in serum, however, was reduced. According to our previous extensive research of GH profiles (Steyn et al., 2011), the GH secretion profile in male mice is characterized by a high-amplitude pulse pattern for less than 30 min every 3–3.5 h with some variability. Therefore, the sampling time with a 1-h interval may not be enough to reflect the GH release pattern of mice compared with a 10-min interval routinely used in this laboratory (Steyn et al., 2011; Huang et al., 2020a).

There were also some indirect evidences to demonstrate the relationship between circadian clock and pulsatile GH secretion. In night workers, the sleep-related GH pulse was lowered, but the reduction was compensated for by the large individual GH pulses occurring during waking periods (Brandenberger and Weibel, 2004). The total GH secretion during the 24 h was constant. Our previous results also found that rotating light disturbed the GH secreted model with more GH pulse numbers and lower GH pulse altitude (Wang et al., 2021). The interaction between pulsatile GH secretion and circadian clock system warrants further research.

Along the aging, circadian rhythm is progressively perturbed and circulating IGF-1 level is reduced, resulting in defects in multiple systematic physiological functions (Tevy et al., 2013). There are also potential interactive pathways between circadian rhythm and IGF-1 levels. Recently, it was revealed that circulating/hepatic IGF-1 levels presented a circadian rhythm in mouse fed *ad libitum*. The level of IGF-1 was higher in the serum during daytime and in the liver during night-time, but lower in the serum during night-time and in the liver during daytime (Patel et al., 2016). Therefore, expression of circadian clock genes may influence the IGF-1 levels. It was reported that *BMAL1* deficient mice had altered circadian rhythm of circulating IGF-1 levels (Patel et al., 2016), and *CRY1* and *2* deficient mice had reduced IGF-1 production (Chaudhari et al., 2017). IGF-1 levels may reset the liver circadian clock (Ikeda et al., 2018).

Due to the difficulty in measuring pulsatile GH secretion, the interaction between the GH-IGF-1 axis and circadian clock warrants further detailed research. Changes of pulsatile GH secretion profile need to be investigated, probably using the animal models with selected circadian clock gene knock-out. Rhythmic changes of circadian clock-related genes by GH demand further investigation in GH-knock-out or mutant animal models. Evidence in observational human studies is requested in future to link animal study to human physiology. The rhythmic changes of circadian clock-related genes in patients with acromegaly or GH deficiency could be helpful. In addition, the pulsatile secretion of GH profiles in patients with sleep deprivation would provide some indirect evidence of circadian change on GH secretion. In summary, the relationship between the GH/IGF-1 axis and the circadian clock system needs to be carefully investigated in the future.

## Conclusion

Both the central circadian clock system and the control center of the GH/IGF-1 axis are located in the hypothalamus and related genes/hormones are expressed/released in circadian rhythm, which regulate metabolism through multi-level interactions. Many people engage in nightshift work with sleep rhythm disorders. The subsequent weight gain, metabolic disorders, and related cardiovascular and cerebrovascular diseases turn into an important public health problem (Scheer et al., 2009; Leproult et al., 2014; Morris et al., 2016). At present, there is no definitive evidence of the interaction between the circadian clock system and the GH/IGF-1 axis and the pathophysiological mechanism causing metabolic disorders. It is utterly necessary to further confirm whether the GH/IGF-1 axis plays a major regulatory role in circadian regulation.

## Author Contributions

LG and CC made substantial contributions to conception and design and revised it critically for important intellectual content. WW, XD, ZH, and QP involved in drafting the manuscript. WW and XD made the figure. All authors contributed to thearticle and approved the submitted version.

## Conflict of Interest

The authors declare that the research was conducted in the absence of any commercial or financial relationships that could be construed as a potential conflict of interest.

## Publisher’s Note

All claims expressed in this article are solely those of the authors and do not necessarily represent those of their affiliated organizations, or those of the publisher, the editors and the reviewers. Any product that may be evaluated in this article, or claim that may be made by its manufacturer, is not guaranteed or endorsed by the publisher.
